# Grafted hPSC-derived GABA-ergic interneurons regulate seizures and specific cognitive function in temporal lobe epilepsy

**DOI:** 10.1038/s41536-022-00234-7

**Published:** 2022-08-01

**Authors:** Dinesh Upadhya, Sahithi Attaluri, Yan Liu, Bharathi Hattiangady, Olagide W. Castro, Bing Shuai, Yi Dong, Su-Chun Zhang, Ashok K. Shetty

**Affiliations:** 1 Institute for Regenerative Medicine, Texas A&M Health Science Center College of Medicine, College Station, TX USA; 2 Department of Molecular and Cellular Medicine, Texas A&M Health Science Center College of Medicine, College Station, TX USA; 3 Research Service, Olin E. Teague Veterans’ Medical Center, Central Texas Veterans Health Care System, Temple, TX USA; 4 Waisman Center, Departments of Neuroscience and Neurology, School of Medicine and Public Health, University of Wisconsin, Madison, WI USA; 5 Present Address: Centre for Molecular Neurosciences, Kasturba Medical College, Manipal Academy of Higher Education, Manipal, Karnataka India; 6 Present Address: Institute of Biological Sciences and Health, Federal Univ of Alagoas (UFAL), Maceio, AL Brazil

**Keywords:** Epilepsy, Stem-cell research

## Abstract

Interneuron loss/dysfunction contributes to spontaneous recurrent seizures (SRS) in chronic temporal lobe epilepsy (TLE), and interneuron grafting into the epileptic hippocampus reduces SRS and improves cognitive function. This study investigated whether graft-derived gamma-aminobutyric acid positive (GABA-ergic) interneurons directly regulate SRS and cognitive function in a rat model of chronic TLE. Human pluripotent stem cell-derived medial ganglionic eminence-like GABA-ergic progenitors, engineered to express hM4D(Gi), a designer receptor exclusively activated by designer drugs (DREADDs) through CRISPR/Cas9 technology, were grafted into hippocampi of chronically epileptic rats to facilitate the subsequent silencing of graft-derived interneurons. Such grafting substantially reduced SRS and improved hippocampus-dependent cognitive function. Remarkably, silencing of graft-derived interneurons with a designer drug increased SRS and induced location memory impairment but did not affect pattern separation function. Deactivation of DREADDs restored both SRS control and object location memory function. Thus, transplanted GABA-ergic interneurons could directly regulate SRS and specific cognitive functions in TLE.

## Introduction

Epilepsy affects 60 million people worldwide. A meta-analysis from 2018 revealed that ~30% of epileptic cases are resistant to drugs^1^, implying ~18 million drug-resistant epilepsy (DRE) cases globally. Although 15 new drugs have been introduced in the past 25 years, the overall proportion of patients with DRE has remained unchanged^2^. Surgical removal of the epileptogenic zone has been one of the major therapeutic options for DRE. However, such surgical resection does not result in efficient long-term seizure control in several cases and can lead to cognitive impairments in many patients^3,4^. Therefore, efficient alternative therapies are urgently needed for controlling seizures and improving cognitive function in patients with DRE. Increased activity of excitatory neurons before the occurrence of seizures has been well demonstrated^5^. Also, the reduced number of various subclasses of gamma-aminobutyric acid positive (GABA-ergic) interneurons^6–8^ and their axon terminals^9^ contributing to seizures are well known. Thus, the replacement of lost GABA-ergic interneurons promises to improve inhibitory synaptic neurotransmission and restore the excitation/inhibition imbalance to prevent seizures in the epileptic brain.

The commencement of several human pluripotent stem cell (hPSC)-based clinical trials worldwide has provided the required initial impetus for the clinical applicability of hPSC-derived cells for several neurological or neurodegenerative disorders. Temporal lobe epilepsy (TLE) is one of the several neurological disorders that could be benefited from GABA-ergic interneuron progenitor cell therapy^10–12^. The efficacy of grafting various cell types such as embryonic hippocampal cells, neural stem cells, lateral and medial ganglionic eminence cells for reducing seizures, improving cognitive function, reducing host interneuron loss, or abnormal plasticity has been demonstrated by multiple previous investigations^13–22^. From a translational point of view, human medial ganglionic eminence (hMGE) cells are the most suitable for treating TLE as a pure population of MGE cells could be easily generated from hPSCs^23^. Moreover, MGE cells readily differentiate into mature GABA-ergic interneurons following grafting, including subtypes expressing parvalbumin (PV), neuropeptide Y (NPY), somatostatin, and others^18,19,22^. Previously, we have demonstrated the ability of transplanted human MGE (hMGE) cells to restrain the evolution of an initial precipitating injury such as status epilepticus into chronic TLE typified by robust spontaneous recurrent seizures (SRS) and cognitive impairments^22^.

The expression of designer receptors exclusively activated by designer drugs (DREADDs) in specific neuronal cell types is a well-established chemogenetic approach to comprehending the function of distinct neural circuits^24–30^. For example, expression of the designer Gi-protein-coupled receptor hM4di (a type of DREADDs) in neurons, a modified form of human M4 muscarinic receptor that can be activated by the clozapine metabolite clozapine-*N*-oxide (CNO), allows silencing of neurons. Such silencing involves activation of hM4di by CNO, triggering the Gi signaling pathway, leading to the opening of potassium channels and the influx of potassium ions. These events decrease the resting membrane potential and the capacity of neurons to depolarize^24^. Studies have shown that the expression hM4Di in the hippocampal excitatory neurons and its subsequent activation by CNO suppressed seizures in the intrahippocampal kainate model of epilepsy^27,31,32^. Moreover, inhibition of dentate gyrus mossy cells via activation of hM4Di expressed on them revealed the role of glutamatergic mossy cells in regulating dentate granule cell activity during epileptogenesis in a pilocarpine model of status epilepticus^33^. Also, the DREADDs strategy has identified the involvement of newly born excitatory dentate granule cells in the development of recurrent excitatory neural circuits in chronic epilepsy^34^. Besides, the expression of hM4di in donor progenitor cells in grafting studies has shown the role of graft-derived dopaminergic neurons in controlling motor function in a model of Parkinson’s disease^35^. Our previous study, employing grafting of hMGE cells expressing hM4Di into the hippocampus shortly after status epilepticus, has demonstrated that graft-derived interneurons that integrate into the hippocampus control seizures in the chronic phase of epilepsy^22^. However, hitherto, no studies have investigated the competence of hMGE cells grafted into the hippocampus after the establishment of chronic epilepsy to regulate seizures and cognitive function by using donor hMGE cells expressing inhibitory DREADDs.

**Fig. 1:**
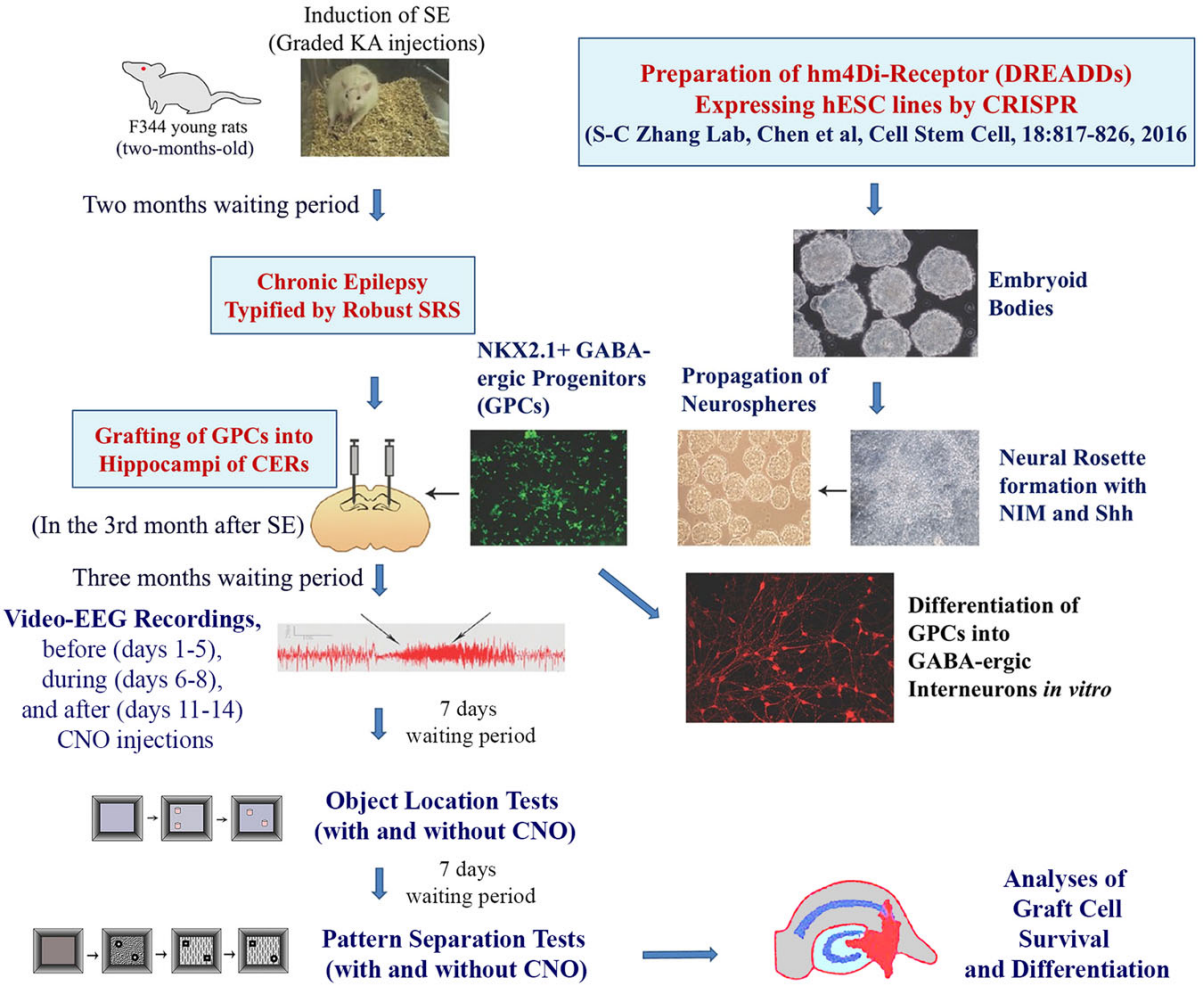
The overall experimental design and the sequence of experiments performed in the study. Status epilepticus (SE) was induced in two-month-old male F344 rats through graded kainic acid injections. Two months later, animals exhibiting chronic temporal lobe epilepsy (TLE) typified by spontaneous recurrent seizures (SRS) were chosen for bilateral grafting of human medial ganglionic eminence (hMGE)-like cells. The hMGE cells were generated from hM4Di-receptor (a designer receptor activated only by designer drugs, DREADDs) expressing human embryonic stem cell (hESC)-line through a directed differentiation protocol. In the fourth month after grafting, rats were implanted with electrodes, and 2 weeks later, continuous video-encephalographic (video-EEG) recordings were taken before (days 1–5), during (days 6–8), and 2 days after (days 11–14) clozapine-*N*-oxide (CNO) treatment. Seven days after completing video-EEG recordings, the animals were probed with the object location tests (OLTs), with and without CNO administration, to assess hippocampus-dependent cognitive function. Seven days after completing the second OLT, the animals were investigated with the pattern separation tests (PSTs), with and without CNO administration, to examine their pattern separation ability. Following behavioral tests, the animals were perfused, and fixed brain tissues were processed to analyze the differentiation and integration of graft-derived cells through dual and triple immunofluorescence methods and confocal imaging.

We generated hMGE progenitors capable of differentiating into mature inhibitory interneurons from human embryonic stem cells (hESCs) expressing stable inhibitory DREADDs (hM4D(Gi)-mCherry) through CRISPR/Cas9 technology^35^. Then, we grafted hMGE progenitors expressing hM4Di into hippocampi of chronically epileptic animals (CERs) to investigate the proficiency of graft-derived inhibitory interneurons in controlling SRS and hippocampus-dependent cognitive functions in a rat model of chronic TLE (Fig. 1). Our results provide direct evidence that hMGE graft-derived interneurons control seizures and regulate specific cognitive behaviors in this prototype of epilepsy. The study also showed that grafted interneurons integrated with DREADDs could be silenced through exogenous drugs when transplantation results in unacceptable side effects.

## Results

### hMGE cell grafting substantially reduced SRS in chronically epileptic rats (CERs)

**Fig. 2:**
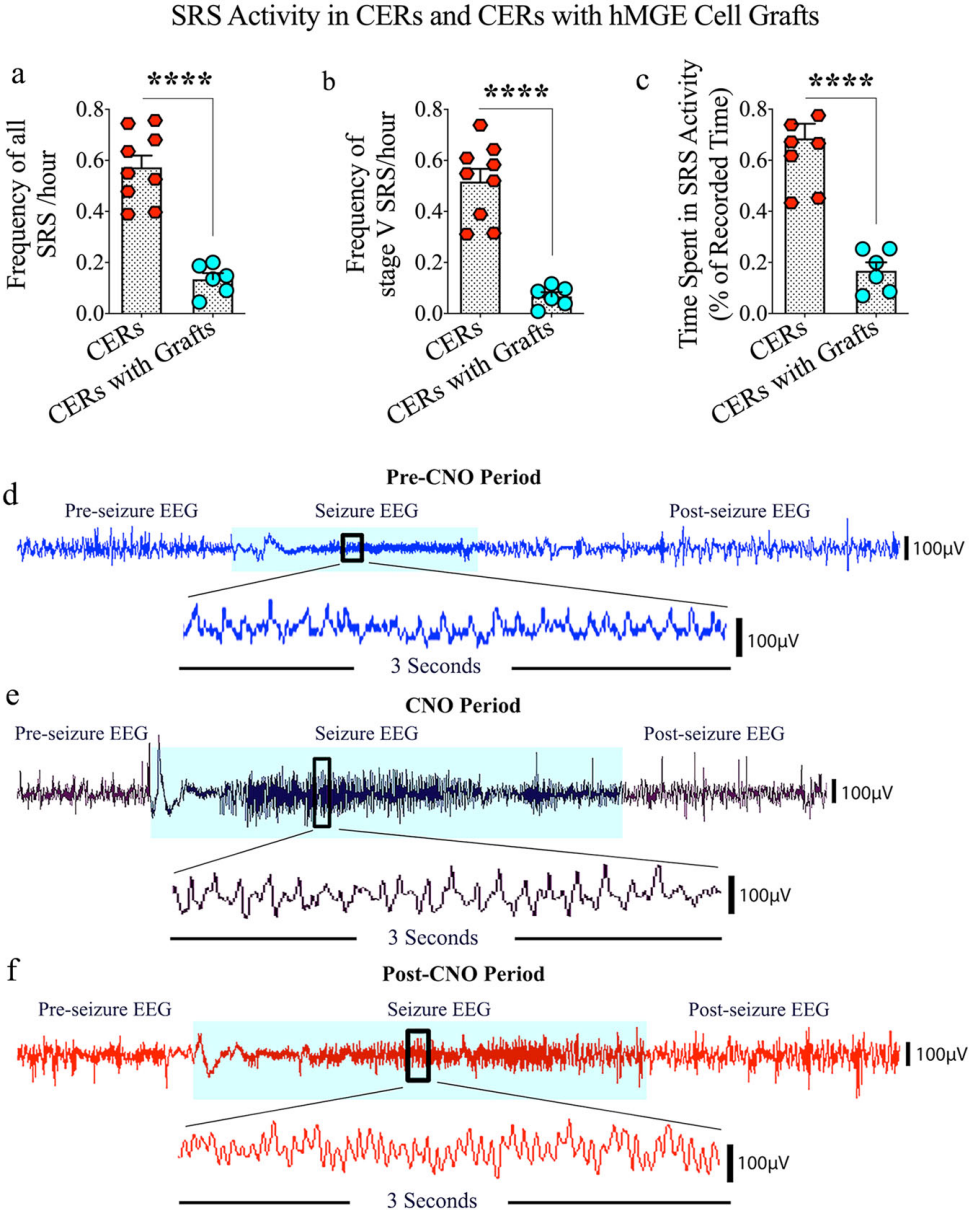
Evaluation of the effects of grafting human medial ganglionic eminence (hMGE) progenitor cells expressing the Gi-protein-coupled receptor hM4Di into the hippocampus of chronically epileptic rats (CERs) on spontaneous recurrent seizure (SRS) activity. Quantification in the 4th month after grafting via continuous video-EEG recordings revealed that compared to the group of CERs receiving no grafts, the group of CERs receiving hMGE cell grafts displayed greatly decreased frequencies of all SRS (**a**) and stage V SRS (**b**). The grafted animals also spent much less time in seizure activity (**c**). **d**–**f** illustrate electroencephalographic (EEG) traces during the pre-clozapine-N-oxide (CNO), CNO, and post-CNO periods. Values in bar charts are presented as mean ± S.E.M. ****p < 0.0001 (unpaired, two-tailed Student’s *t* test).

The effect of grafting hMGE progenitors expressing the Gi-protein-coupled receptor hM4Di into the hippocampus of CERs was evaluated on SRS activity in the fourth month after grafting through continuous video-electroencephalographic (video-EEG) recordings (Fig. 1). The CERs receiving grafts were immunosuppressed with daily cyclosporine A injections (10 mg/kg) starting two days before grafting and continuing until the experimental endpoint to avoid transplant rejection. Ungrafted control CERs also received the same regimen of cyclosporine injections to identify any cyclosporine-induced effects on seizures. The total numbers of SRS and stage V-SRS and the total time spent in seizure activity were measured. Compared to ungrafted CERs, grafted CERs displayed substantial reductions in the number of SRS/hour (76% reduction, *p* < 0.0001, unpaired, two-tailed Student’s *t* test Fig. 2a), number of stage V-SRS/hour (87% reduction, *p* < 0.0001, unpaired, two-tailed Student’s *t* test, Fig. 2b), and the total time spent in seizure activity (76% reduction, *p* < 0.0001, unpaired, two-tailed Student’s *t* test, Fig. 2c). Thus, grafting of hPSC-derived hMGE progenitors into the hippocampus in the chronic phase of TLE significantly reduced both frequency and intensity of SRS.

### SRS activity escalated in CERs when graft-derived GABA-ergic interneurons were silenced through CNO-mediated DREADDs activation

**Fig. 3:**
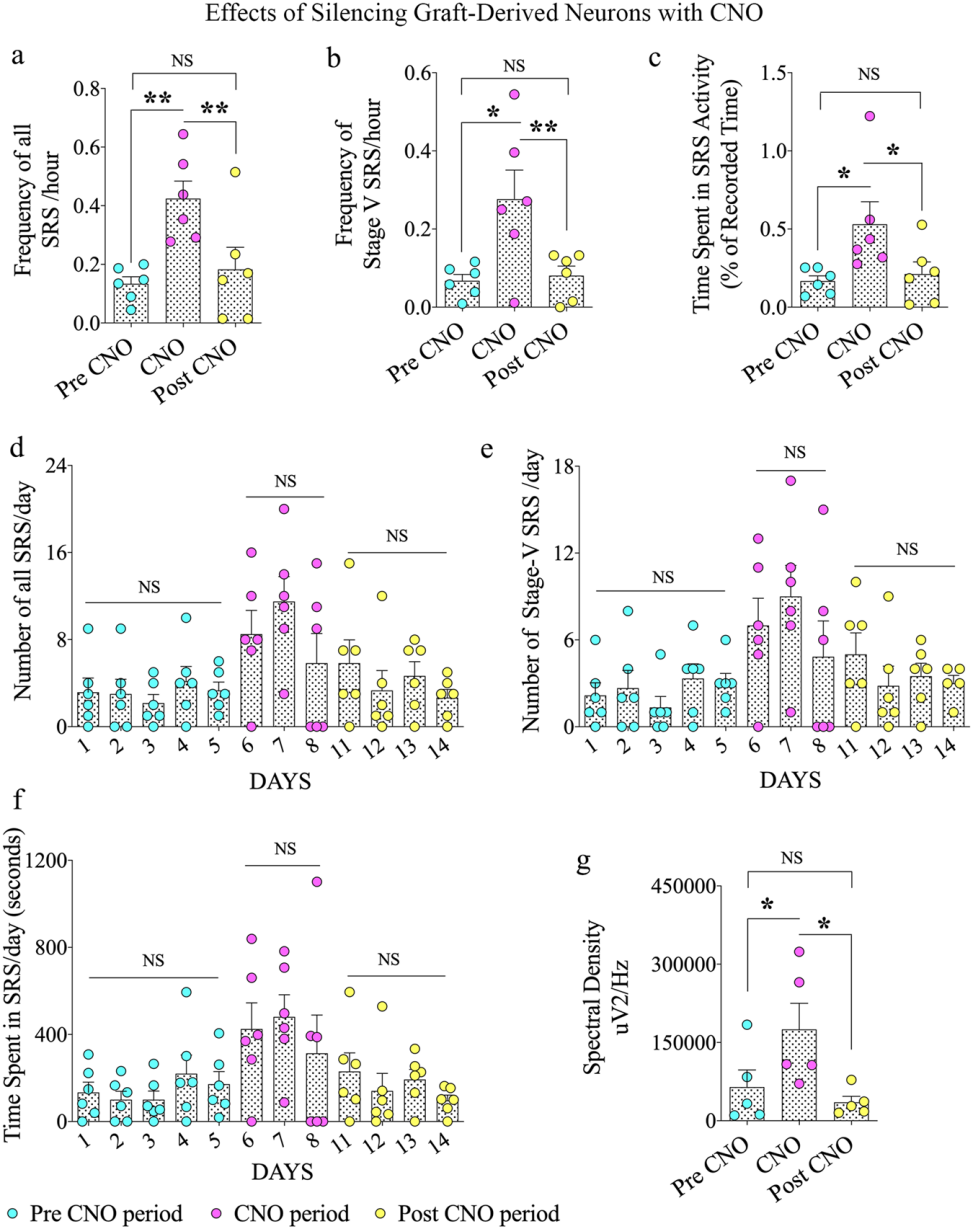
Effects of silencing graft-derived neurons with clozapine-N-oxide (CNO) on spontaneous recurrent seizure (SRS) activity in chronically epileptic rats. The bar charts **a**–**c** compare all SRS and stage V SRS frequencies and times spent in SRS activity (% of recorded time) during pre-CNO, CNO, and post-CNO periods. The bar charts **d**–**f** compare all SRS and stage V SRS frequencies and times spent in SRS activity during the pre-CNO (days 1–5), CNO (days 6–8), and post-CNO (days 11–14) periods. The bar chart **g** compares the average electroencephalographic (EEG) power (i.e., spectral density) recorded in interictal periods during pre-CNO, CNO, and post-CNO phases. Values in bar charts are presented as mean ± S.E.M. *p < 0.05; **p < 0.01; NS, non-significant (one-way ANOVA with Newman–Keuls multiple comparisons test).

Continuous video-EEG recordings before (days 1–5), during (days 6–8), and 2 days after (days 11–14) silencing the graft-derived GABA-ergic interneurons through CNO injections evaluated the influence of graft-derived interneurons in controlling SRS activity in CERs receiving grafts. Examples of EEG traces during the pre-CNO, CNO, and post-CNO periods are illustrated (Fig. 2d–f). Because the action of CNO is expected to last 2–3 h after each administration and to avoid effects associated with its accumulation due to repeated administration, we administered CNO once every 8 h to activate DREADDs. Also, we employed 2 days of washout period to avoid any trace amounts of CNO interfering with the post-CNO results. Silencing of graft-derived neurons substantially escalated SRS activity in CERs compared to the extent of SRS activity before CNO administration (Fig. 3a–c). Overall, one-way analysis of variance (ANOVA) with the Newman–Keuls multiple comparison tests revealed that there was a 1.4–9.7-fold increase in the frequency of all SRS (*p* < 0.01, Fig. 3a), 1.2–6.4-fold increase in the frequency of stage V-SRS (*p* < 0.05, Fig. 3b), and 1.3–6.2-fold increase in the total time spent in SRS activity (*p* < 0.05, Fig. 3c). Then, the effect of CNO washout on SRS activity was evaluated two days after the last CNO injection. All parameters of SRS activity were restored to pre-CNO levels. One-way ANOVA with Newman-Keuls multiple comparison tests showed that compared to the CNO period, the frequencies of SRS and stage V-SRS were reduced by 57–71% (*p* < 0.01, Fig. 3a, b), and the time spent in seizure activity was reduced by 60% (*p* < 0.05, Fig. 3c).

We also evaluated the seizure parameters/day in the pre-CNO (days 1–5), CNO (days 6–8), and post-CNO periods (days 11–14; Fig. 3d–f) using one-way ANOVA with the Newman–Keuls multiple comparison tests. In CERs receiving grafts, the total SRS and stage-V SRS/day and the time spent in SRS activity/day were lower in the pre-CNO period. There was no difference in seizure activity over five days in this phase (*p* > 0.05). The administration of CNO enhanced the total SRS and stage-V SRS/day and the time spent in SRS/day. Furthermore, comparable seizure activity was seen over the 3-day CNO period (*p* > 0.05; Fig. 3d–f). The total number of all SRS on day 7 in the CNO period was higher than all SRS on pre-CNO days 1–5 (*p* < 0.05; Fig. 3d). Also, the number of stage-V SRS on day 7 in the CNO period was significantly higher than stage-V SRS recorded on pre-CNO days 1 and 3 (*p* < 0.05; Fig. 3e). Notably, all parameters of seizures/day declined in the post-CNO period after two days of CNO washout (Fig. 3d–f). Also, there was no difference in seizure activity during the four-day post-CNO period (*p* > 0.05). The total numbers of all SRS on days 12–14 in the post-CNO period were significantly lower than all SRS recorded on day 7 in the CNO period (*p* < 0.05; Fig. 3d). Additionally, all parameters of seizures were comparable between pre-CNO (days 1–5) and post-CNO (days 11–14) periods (*p* > 0.05; Fig. 3d–f), implying that the inhibitory function of graft-derived interneurons is restored after the CNO washout period.

Furthermore, we performed spectral analysis of EEG activity in interictal periods by measuring randomly chosen thirty-minute duration interictal segments devoid of noise signals (6–10 segments/animal, *n* = 5/group). One-way ANOVA with the Newman-Keuls multiple comparison tests revealed that compared to the pre-CNO period, the average EEG power enhanced in the CNO period (*p* < 0.05, Fig. 3g). However, following the CNO washout, the EEG power declined substantially (*p* < 0.05, Fig. 3g). Also, the percentage of β waves is significantly reduced in the CNO period compared to the pre-CNO period (mean ± S.E.M., pre-CNO period 17.5 ± 3.0; CNO period, 8.7 ± 1.2; *p* < 0.05) but increased following CNO washout (11 ± 1.9). Overall, in addition to enhancing the frequency and intensity of SRS, silencing graft-derived GABA-ergic interneurons through CNO injections resulted in enhanced interictal activity, which subsequently waned after the CNO washout.

**Fig. 4:**
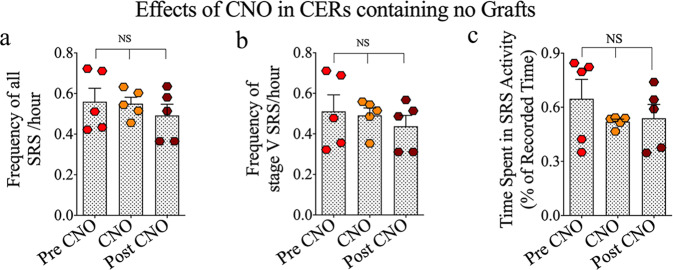
Effects of clozapine-N-oxide (CNO) on spontaneous recurrent seizure (SRS) activity in chronically epileptic rats receiving no grafts. The bar charts **a**–**c** compare all SRS and stage V SRS frequencies and times spent in SRS activity (% of recorded time) during the pre-CNO, CNO, and post-CNO periods in CERs receiving no grafts. Values in bar charts are presented as mean ± S.E.M. NS, non-significant (one-way ANOVA with Newman–Keuls multiple comparisons test).

Next, to examine the direct effect of CNO on SRS activity, we measured SRS activity in CERs that did not receive grafts with CNO administration. One-way ANOVA with the Newman–Keuls multiple comparison tests demonstrated that the frequencies of all SRS and stage V-SRS and the time spent in SRS activity remained comparable across pre-CNO, CNO administration, and post-CNO periods (*p* > 0.05, Fig. 4a–c). Thus, in CERs receiving hMGE cell grafts, SRS activity increased when graft-derived GABA-ergic interneuron function was blocked, implying the direct involvement of graft-derived interneurons in seizure control. Furthermore, CNO alone did not affect SRS activity, as CNO administration in CERs receiving no grafts did not change all SRS and stage V-SRS frequencies or the time spent in SRS activity.

### Grafting of hMGE cells into hippocampi improved hippocampus-dependent cognitive function in CERs

**Fig. 5:**
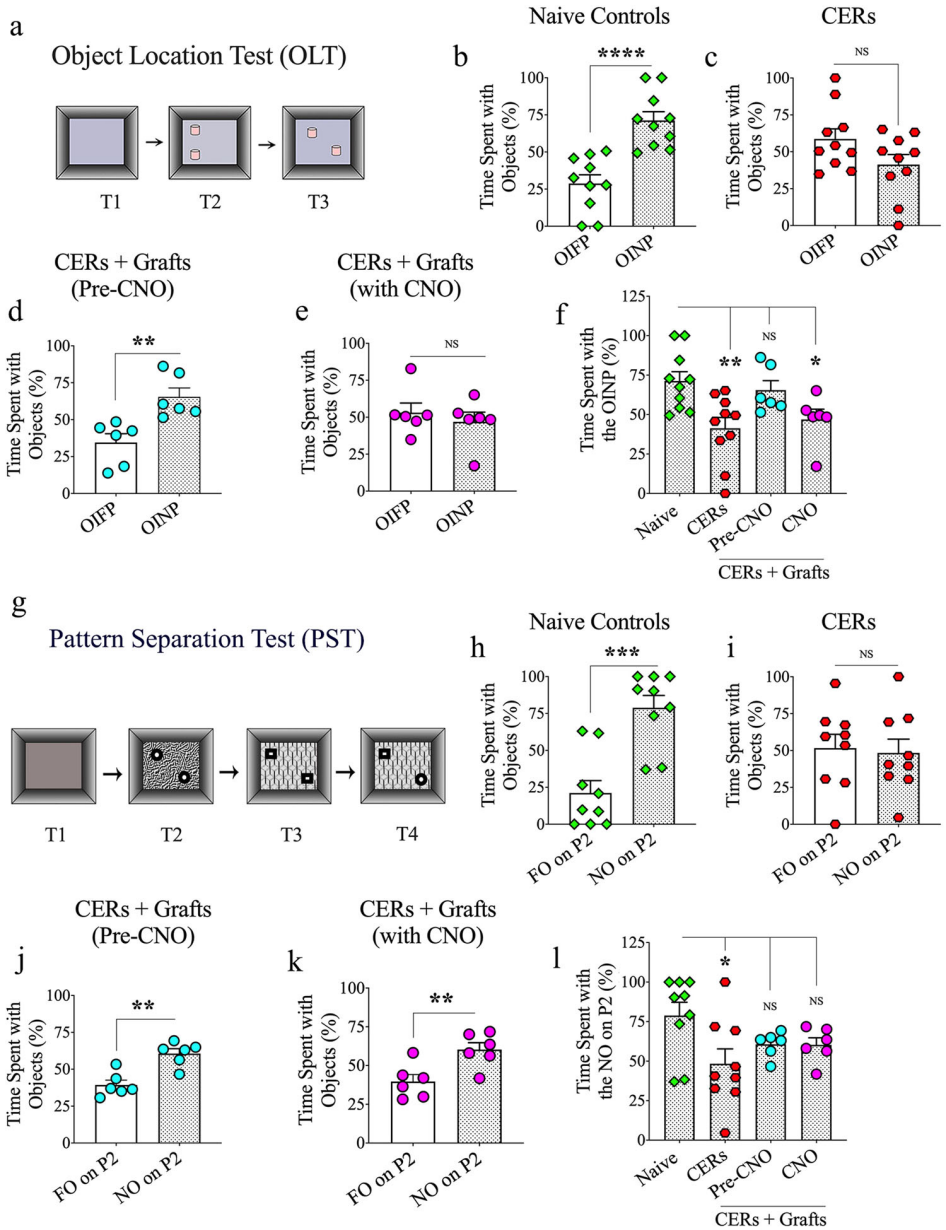
Effects of grafting human medial ganglionic eminence (hMGE) progenitor cells expressing the Gi-protein-coupled receptor hM4Di into the hippocampus of chronically epileptic rats (CERs) on cognitive function. **a** depicts the various trials involved in an object location test (OLT). The bar charts in **b**–**e** compare percentages of time spent with the object in the familiar place (OIFP) and the object in the novel place (OINP) in naive control rats (**b**), chronically epileptic rats (CERs; **c**), and CERs with hMGE grafts before and during the clozapine-N-oxide (CNO) treatment (**d**, **e**). The bar chart in **f** compares the time spent with the OINP across the four groups with ANOVA. Object location memory was impaired in CERs with no grafts and CERs with grafts when graft-derived interneurons were silenced. **g** shows the various trials involved in a pattern separation test (PST). The bar charts in **h**–**k** compare percentages of time spent with the familiar object on pattern 2 (FO on P2) and the novel object on pattern 2 (NO on P2) in naive control rats (**h**), CERs (**i**), and CERs with hMGE grafts before and during the clozapine-N-oxide CNO treatment (**j**, **k**). The bar chart in **l** compares the time spent with the NO on P2 across the four groups with ANOVA. Note that pattern separation ability was impaired in CERs with no grafts. However, CERs with grafts displayed pattern separation ability even when the graft-derived interneurons were silenced with CNO. Values in bar charts are presented as mean ± S.E.M. *p < 0.05, **p < 0.01, ***p < 0.001, ****p < 0.0001, NS, non-significant (**b**–**e**, **h**–**k**, unpaired, two-tailed Student’s *t* test; **f**, **l**, one-way ANOVA with Newman–Keuls multiple comparisons test).

We employed an object location test (OLT) to examine the cognitive ability of animals to detect subtle changes in their immediate environment (Fig. 5a), a function linked to normal network activity in the hippocampus^36,37^. In OLT, the animals with altered hippocampal circuitry/dysfunction consistently show an inability to detect minor alterations in the environment. Naive control rats recognized the change that occurred in the position of one of the objects by exploring the object in the novel place (OINP) for significantly greater periods than the object that remained in the familiar place (OIFP, *p* < 0.0001, unpaired, two-tailed Student’s *t* test, Fig. 5b) in trial-3 (T3). In contrast, CERs receiving no grafts showed impaired cognitive function by spending nearly equal amounts of their object exploration time with the OINP and the OIFP (*p* > 0.05, unpaired, two-tailed Student’s *t* test, Fig. 5c). Notably, CERs receiving hMGE cell grafts behaved similarly to naive control rats by showing a greater affinity for the OINP than the OIFP (*p* < 0.01, unpaired, two-tailed Student’s *t* test, Fig. 5d). These results suggest that grafting of hPSC-derived hMGE cells into the hippocampus could alleviate chronic epilepsy-related object location memory impairment.

### Graft-derived GABA-ergic interneurons directly influenced the object location memory task in CERs

To investigate the role of graft-derived GABA-ergic interneurons in the object location memory task, we silenced the transplant-derived DREADDs expressing interneurons through CNO administration and performed the OLT with new objects. With the silencing of transplant-derived interneurons, CERs lost their ability to distinguish the OINP from the OIFP, which was evident from their exploration of OINP and OIFP for almost equal periods (*p* > 0.05, unpaired, two-tailed Student’s *t* test, Fig. 5e). The parameters such as total object exploration times, distances traveled, or movement velocities were comparable between the pre-CNO and CNO periods (data not illustrated). Comparison of the time spent with the OINP across groups (naive, CERs, CERs + grafts in the pre-CNO and CNO periods) using one-way ANOVA with the Newman-Keuls multiple comparison tests revealed impaired object location memory in CERs with no grafts, and CERs with grafts when graft-derived interneurons were silenced (Fig. 5f). However, in the absence of CNO, CERs with grafts displayed similar object location memory as naive control rats. Thus, graft-derived GABA-ergic interneurons in CERs directly influenced the object location memory function, a hippocampus-dependent cognitive task.

### Grafting of hMGE cells into hippocampi improved pattern separation function in CERs

The pattern separation test (PST) examines proficiency in discriminating similar experiences by storing similar representations in a non-overlapping manner and is linked to the dentate gyrus and adult hippocampal neurogenesis^38,39^. For this test, the movement of each rat was investigated in an open field with four successive trials (5 min each), separated by 30-min intervals. The first three trials successively involved the exploration of an open field apparatus (T1), a type of identical objects placed on a floor pattern 1 (P1; T2), and the second type of identical objects placed on a floor pattern 2 (P2; T3). In T4, the animal explored objects on P2, with one of the objects from T3 replaced with an object from T2. The object from T2 is a novel object on pattern 2 (NO on P2), whereas the object retained from T3 is a familiar object on P2 (FO on P2) (Fig. 5g). Naive control animals displayed a greater propensity to explore the NO on P2 than the FO on P2 in T4 (*p* < 0.001, unpaired, two-tailed Student’s *t* test, Fig. 5h). CERs receiving no grafts displayed a pattern separation deficit, which was evident from their lack of interest in exploring the NO on P2 for higher periods than the FO on P2 (*p* > 0.05, unpaired, two-tailed Student’s *t* test, Fig. 5i). In contrast, CERs receiving grafts showed similar behavior as naive control animals by displaying a greater propensity to explore the NO on P2 than the FO on P2 (*p* < 0.01, unpaired, two-tailed Student’s *t* test, Fig. 5j). Thus, grafting of hPSC-derived hMGE cells into the hippocampus alleviated chronic epilepsy-induced pattern separation dysfunction.

### Graft-derived GABA-ergic interneurons did not control the pattern separation function in CERs receiving grafts

To determine whether graft-derived interneurons played a role in restoring the pattern separation function in CERs, we silenced the transplant-derived DREADDs expressing interneurons through CNO administration and performed the PST with new objects and floor patterns. With the silencing of transplant-derived interneurons, CERs did not lose their ability to distinguish the NO on P2 from the FO on P2, which was evident from their exploration of the NO on P2 for higher periods than FO on P2 (*p* < 0.01, unpaired, two-tailed Student’s *t* test, Fig. 5k). Furthermore, the parameters such as total object exploration times, distances traveled, or movement velocities were comparable between the pre-CNO and CNO periods (data not illustrated). Comparison of the time spent with the NO on P2 across groups (naive, CERs, CERs + grafts in the pre-CNO and CNO periods) using one-way ANOVA with the Newman-Keuls multiple comparison tests revealed impaired pattern separation function in CERs with no grafts, but not in CERs with grafts even when graft-derived interneurons were silenced (Fig. 5l). Thus, CERs with grafts displayed similar pattern separation ability as naive control rats in the absence and presence of CNO, implying that graft-derived GABA-ergic interneurons in CERs did not directly influence the pattern separation function.

### Cells derived from hMGE cell grafts displayed robust survival

Stereological quantification of HNA+ cells per hippocampus revealed that the overall graft cell yield is >800,000 cells/hippocampus (mean ± S.E.M = 886,266 ± 55,967, *n* = 4). Since the graft cell yield per hippocampus was higher than the number of cells initially injected (~300,000 live cells in 3 grafts, ~100,000 cells/graft), the results implied that the grafted progenitors divided a few times after grafting as some donor cells likely die during transplantation.

### All graft-derived interneurons expressed DREADDs

**Fig. 6:**
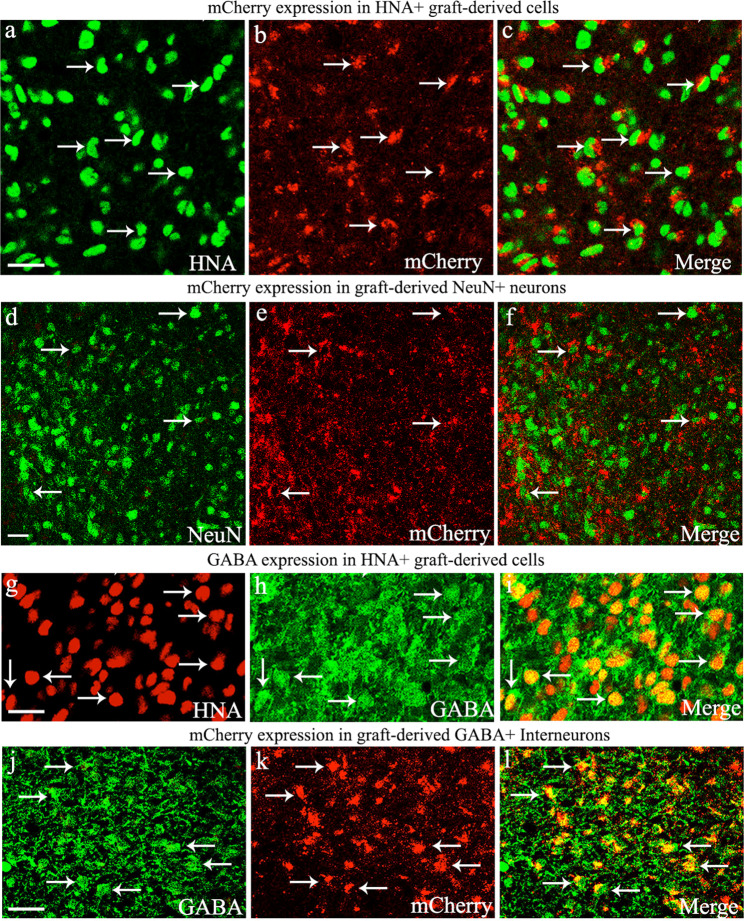
Evaluation of Gi-protein-coupled receptor hM4Di expression (with mCherry reporter) in human nuclear antigen-positive (HNA+) cells, neuron-specific nuclear antigen-positive (NeuN+) neurons, and gamma-aminobutyric acid-positive (GABA+) interneurons derived from human medial ganglionic eminence progenitor cell grafts in the hippocampus of chronically epileptic rats. Note that mCherry is displayed in virtually all HNA+ graft-derived cells (**a**–**c**), NeuN+ neurons (**d**–**f**), and GABA-ergic interneurons (**j**–**l**). **g**–**i** demonstrate that a vast majority (mean ± S.E.M, 80.8 ± 1.1%) of HNA+ graft-derived cells differentiated into GABA-ergic interneurons. Scale bars: **a**–**l**, 20 µm.

To confirm DREADD expression in transplant-derived cells, we performed immunofluorescence studies on tissue sections through the hippocampus to visualize human nuclear antigen (HNA, a marker of grafted human cells) and neuron-specific nuclear protein (NeuN, a marker of neurons). Confocal microscopic analyses of HNA and mCherry (the reporter of DREADD expression) revealed that virtually all HNA+ cells in grafts expressed DREADDs (Fig. 6a–c). Similar analysis of NeuN and mCherry showed that all neurons within grafts expressed DREADDs (Fig. 6d–f). The hESC line employed in the study was built by inserting a construct of DREADD and mCherry separated by 2 A. Furthermore, the expression of DREADD and mCherry in the cell line is under the control of the universal CAG promoter, and hence mCherry is expressed stably in all cells. Earlier grafting studies have demonstrated similar results using this cell line^35,40^.

**Fig. 7:**
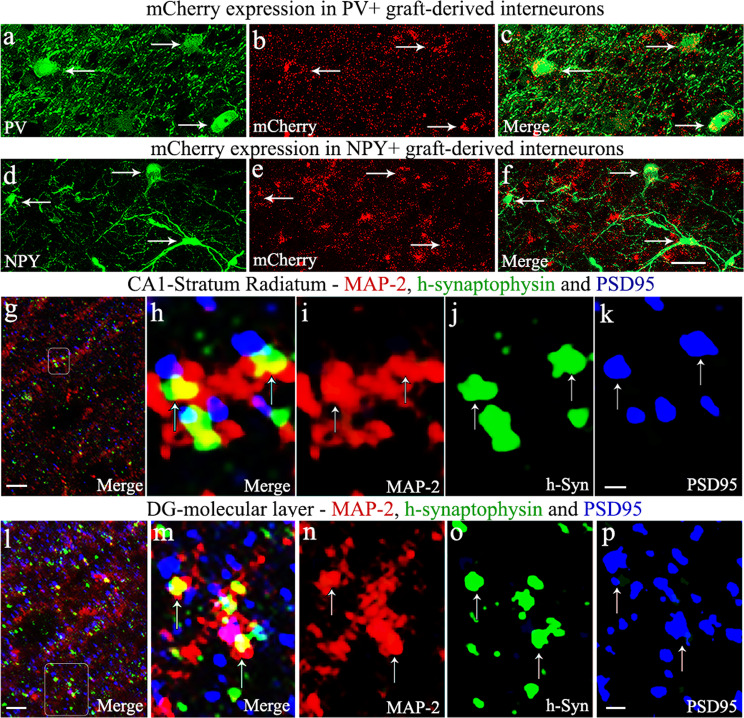
Graft-derived interneurons expressed hM4Di and formed putative synapses with host neurons. Gi-protein-coupled receptor hM4Di expression (with mCherry reporter) in parvalbumin (PV) and neuropeptide Y (NPY) expressing interneurons derived from human medial ganglionic eminence progenitor cell grafts in the hippocampus of chronically epileptic rats (**a**–**f**), and putative synapse formation between graft-derived axons and host neurons (**g**–**p**). Note that mCherry is apparent in PV and NPY+ interneurons derived from graft-derived cells (**a**–**f**). **g**, **l** illustrate putative synapse formation between graft-derived presynaptic boutons (green colored structures expressing human synaptophysin (hSyn) and the host postsynaptic density protein 95 (PSD95, red particles) elements on microtubule-associated protein-2 (MAP-2) positive dendrites (blue) in the host CA1 stratum radiatum (**g**) and the dentate gyrus molecular layer (**l**). **h**, **m** are magnified views of boxed regions in **g**, **l**, respectively. **i**–**k**, **n**–**p** illustrate MAP-2, hSyn, and PSD95 elements in red, green, and blue channels. Scale bars: **a**–**f**, 20 µm; **g**, **l**, 5 µm; **i**–**k**, **n**–**p**, 0.5 µm.

Next, we determined the differentiation of graft-derived cells into NeuN+ neurons or GABA+ interneurons through HNA and NeuN, or HNA and GABA dual immunofluorescence and *Z*-section analysis in a confocal microscope. Such quantification demonstrated that ~85% of HNA+ expressed NeuN (mean ± S.E.M = 85.2 ± 1.2, *n* = 6) and ~81% of HNA+ cells expressed GABA (mean ± S.E.M = 80.8 ± 1.1%, *n* = 6). Examples of hMGE cells differentiating into GABA-ergic interneurons are illustrated (Fig. 6g–i). The overall differentiation is consistent with our earlier grafting study using hiPSC-derived MGE cells as donor cells in an SE model^22^. Next, to confirm the expression of DREADDs in graft-derived GABA-ergic interneurons, we examined mCherry expression in these interneurons. Virtually all GABA-ergic interneurons expressed mCherry (Fig. 6j–l). In addition, transplanted hMGE cells also differentiated into subclasses of GABA-ergic interneurons expressing PV or NPY, which also displayed DREADDs (Fig. 7a–f). These results suggest that CNO administration could block the function of graft-derived interneurons because of their robust expression of DREADDs.

### Graft-derived neurons formed putative synaptic connections with host CA1 pyramidal neurons and dentate granule cells

Enhanced frequency and intensity of SRS following silencing of graft-derived GABA-ergic interneurons expressing DREADDs implied connectivity between hMGE graft-derived GABA-ergic interneurons and the host neurons. To confirm this, we employed *Z*-section analyses in a confocal microscope of brain tissue sections through the hippocampus processed for triple immunofluorescence to localize the human-specific synaptophysin (hSyn, the presynaptic protein in graft-derived neurons), postsynaptic density protein-95 (PSD-95), and microtubule-associated protein-2 (MAP-2) in soma and dendrites of host neurons. Such analysis suggested the formation of putative synaptic contacts by graft-derived neurons on the dendrites of host CA1 pyramidal neurons in the stratum radiatum (Fig. 7g) and dentate granule cells in the molecular layer (Fig. 7l). Magnified views showing the possible contacts between the presynaptic component derived from graft-derived interneurons (h-Syn+ structures in green) and the host postsynaptic component (PSD95+ structures in blue) on the dendrites of CA1 pyramidal neurons and dentate granule cells (in red) are illustrated (Fig. 7h–k, m–p). In addition, hSyn+ structures were also seen on the soma of dentate granule cells. Thus, transplanted GABA-ergic interneurons appeared to have integrated synaptically with the host neurons in the dentate gyrus and the CA1 subfield. Such synaptic connectivity likely explains the control of seizures and object location memory task by transplant-derived GABA-ergic interneurons.

## Discussion

Spontaneous recurrent seizures and significant cognitive impairments characterize chronic TLE. Substantial loss or altered function of GABA-ergic interneurons have been suggested to contribute to SRS occurrence in TLE^6,8,10^. Previous studies in several rodent models of TLE have demonstrated that transplantation of GABA-ergic progenitors into the hippocampus is an efficient approach for repressing seizures and improving cognitive function^15,18–20^. However, mechanisms underlying seizure suppression or improved cognitive function after grafting inhibitory interneurons in TLE models have been primarily speculative^10^. A precise mechanism by which graft-derived neurons repress seizures is not a critical requirement for clinical translation of cell therapy when functional efficacy is consistently observed without adverse effects. Nonetheless, comprehending mechanisms underlying graft-mediated benefits would aid in further refining the grafting approach, such as defining an appropriate dose for enhancing the beneficial effects on a long-term basis. By transplanting hMGE progenitors generated from hPSCs expressing DREADDs (Gi-protein-coupled receptor hM4Di), the current study provided direct evidence of the involvement of transplant-derived GABA-ergic interneurons in seizure control and the improved hippocampus-dependent cognitive function. Furthermore, the grafting paradigm employed in the study demonstrated that exogenous administration of the designer drug CNO could control the activity of transplant-derived inhibitory neurons in the hippocampus. Such an approach has implications when transplantation leads to adverse effects on brain function.

Hyperactivity of excitatory glutamatergic neurons or declined inhibitory neurotransmission resulting from either the loss or inactivity of inhibitory GABA-ergic interneurons can trigger seizures. Persistent excitatory-inhibitory imbalance or uncontrolled excitatory neuron activity could lead to the occurrence of SRS^5,41^. Thus, strategies/drugs reducing the activity of excitatory glutamatergic neurons or enhancing the inhibitory GABA-ergic neurotransmission have the promise to control seizures. Indeed, a recent study has demonstrated that seizures could be reduced by chemogenetic silencing of the hyperactive excitatory glutamatergic granule cells in the dentate gyrus^34^. Furthermore, GABA-ergic interneuron progenitor cell grafting in SE or TLE models has primarily resulted in reduced SRS activity^15,18–20,22^, but one study in a mouse model of TLE reported no effects of hMGE-like cell grafting on seizure activity^42^. Substantial reductions in all SRS and stage V-SRS and the total time spent in seizure activity in CERs receiving grafts compared to CERs receiving no grafts in the present study further validated the efficacy of grafting MGE progenitors derived from hPSCs for seizure control in TLE. The purity of donor hMGE cells is likely one of the most critical aspects of successful seizure control after grafting into the epileptic brain. In this study, we used DREADDs integrated hMGE cells generated through directed differentiation of hPSCs using high concentrations of sonic hedgehog following neuroepithelial induction^23^, which resulted in the derivation of >90% NKX2.1+ MGE progenitor cells from hPSCs. Furthermore, graft-derived cells survived robustly, and most graft-derived cells (>80%) expressing DREADDs differentiated into GABA-ergic interneurons.

The regulation of excitatory neurons expressing DREADDs in the brain through exogenous CNO administration has been reported in epilepsy models^32,34^. The current study demonstrated that graft-derived GABA-ergic interneuron function could be silenced through CNO administration. Silencing of DREADDs expressing graft-derived interneurons resulted in increased seizure activity compared to seizures in the pre-CNO period. Such results implied that transplant-derived GABA-ergic interneurons directly restrained the generation of SRS. We validated this concept further by recording and evaluating SRS activity after the CNO washout period. Remarkably, CNO washout mediated deactivation of DREADDs in graft-derived interneurons reduced SRS activity to levels seen in the pre-CNO period, which confirmed the direct seizure control by graft-derived GABA-ergic interneurons. Immunofluorescence and confocal microscopic studies confirmed the robust expression of DREADDs by graft-derived interneurons. Moreover, by visualizing graft-derived presynaptic terminals and PSD95 boutons on the MAP-2+ soma and dendrites of host CA1 pyramidal and dentate granule cells, the study implied that graft-derived neurons have likely established synaptic contacts with the host neurons in the dentate gyrus and the CA1 subfield. Connectivity between the transplant-derived inhibitory interneurons and the host hyperactive excitatory neurons in the dentate gyrus and the CA1 subfield have likely prevented amplification of excitatory signals leading to seizure control in CERs receiving hMGE progenitor cell grafts.

Cognitive impairment is one of the conspicuous co-morbidities of chronic TLE. GABA-ergic interneurons and their subtypes are believed to play essential roles in multiple neurocognitive functions^43^. Previous studies have demonstrated improved cognitive and memory function in SE or TLE models or traumatic brain injury-induced seizure models following intrahippocampal grafting of MGE progenitors obtained from rodent embryonic brain or hPSCs^18,20,22,42,44^. The current study showed improvements in cognitive measures such as the CA1-dependent object location memory and the dentate gyrus-dependent pattern separation function in CERs receiving hMGE progenitor cell grafts. Remarkably, silencing transplant-derived GABA-ergic interneurons through CNO administration resulted in object location memory impairment, implying the direct influence of transplant-derived GABA-ergic interneurons in a location memory task. However, similar silencing did not affect the improved pattern separation function seen in CERs receiving hMGE progenitor cell grafts. The results underscore that an improved object location memory function in CERs receiving hMGE progenitor cell grafts is likely a result of synaptic integration of graft-derived interneurons and the host neurons in the CA1 subfield. On the other hand, comparable results of the pattern separation test in the pre-CNO and CNO periods in CERs with grafts suggest that pattern separation function is independent of the synaptic integration between graft-derived neurons with the host neurons in the dentate gyrus. It is plausible that improved pattern separation function in CERs receiving grafts is a consequence of multiple indirect factors induced by hMGE progenitor cell grafting during the several months of the post-grafting survival period. These may include increased production of neurotrophic factors and neuropeptides by host and graft-derived neurons, dampening the hyperactivity of host excitatory dentate granule cells for prolonged periods and/or improved hippocampal neurogenesis. Increased neurotrophic factors and neuropeptides could also enhance endogenous neurogenesis^45–50^. Since none of these parameters were measured in this study, additional studies are critical in the future to comprehend mechanisms underlying the differential effects of silencing of graft-derived GABA-ergic interneurons on OLT vis-à-vis PST.

In summary, the study provided direct evidence that graft-derived GABA-ergic interneurons can control SRS occurrence in a model of chronic TLE. Furthermore, GABA-ergic interneurons directly influenced the hippocampus-dependent object location memory function but not the gross pattern separation task.

## Methods

### Generation of chronically epileptic rats

Rats with chronic TLE were generated through induction of status epilepticus (SE) via graded intraperitoneal injections of kainic acid in 2-month-old male Fischer 344 rats^51,52^. The development of chronic epilepsy in the third month after SE was confirmed by the direct observation of spontaneous recurrent seizures (SRS) in these rats for at least 60 h over 2–3 weeks. From this cohort, chronically epileptic rats (CERs) having a similar range of SRS were randomly assigned to 3 different groups, a group receiving no treatment (CERs, mean SRS = 0.24 ± 0.03, *n* = 9) and a group receiving bilateral grafting of hPSC-derived hMGE cells expressing hM4Di DREADDs (CERs with grafts, mean SRS = 0.25 ± 0.03, *n* = 6) and a group receiving clozapine N-oxide (CNO), the drug activating DREADDs (CERs with CNO, mean SRS = 0.24 ± 0.03, *n* = 5). Additionally, a group of age-matched naive control rats (*n* = 9) was also included to compare behavioral results. All rats were obtained from Harlan and housed in an environmentally controlled room with a 12:12-h light–dark cycle, and ad libitum food and water were provided. All experiments performed were approved by the institutional animal care and use committee of the Texas A&M Health Sciences Center and Central Texas Veterans Health Care System. Figure 1 illustrates the type and timeline of various experiments.

### Construction of donor plasmid

Human codon-optimized Streptococcus pyogenes wild-type Cas9 (Cas9-2A-GFP) and sgRNA T2 were obtained from Addgene (plasmid #44719, plasmid#41818)^53,54^. To generate AAVS1-pur-CAG-EGFP donor plasmid, we replaced the hrGFP gene in the AAVS1-pur-CAG-hrGFP plasmid (Addgene plasmid #52344)^55^ with EGFP gene and inserted woodchuck hepatitis post-transcriptional regulatory element (WPRE) and human growth hormone (hGH) Poly A into the 3’ terminal of EGFP gene to obtain AAVS1-pur-CAG-EGFP donor plasmid. We next amplified hM4Di-mCherry cDNA by PCR from AAV-DIO-hM4Di-mCherry plasmid (a gift from Dr. Bryan L. Roth), respectively. hM4Di-mCherry was inserted into the AAVS1-pur-CAG-EGFP donor plasmid to replace EGFP to get the AAVS1-pur-CAG-hM4Di-mCherry donor plasmid.

### Generation of NKX2.1+ MGE progenitors from hPSCs expressing DREADDs

Human PSCs (WA09 hESC line from WiCell, passages 20–40) were used to generate hM4Di expressing hMGE cells. Institutional Biosafety Committees at the University of Wisconsin and Texas A&M University have approved research on human PSC lines from WiCell. Briefly, ROCK inhibitor-treated human PSCs were lifted with dispase to produce single cells. These cells are electroporated with a cocktail containing Cas9 plasmid (15 µg) sgRNA T2 plasmid (15 µg), and donor plasmid (30 µg) in 500 ml of electroporation buffer containing HEPES using the Gene Pulser Xcell System (Bio-Rad) at 250 V, 500 mF in a 0.4 cm cuvette. Consequently, cells were plated onto the MEF feeder layer in 6-well plates, and colonies were picked up individually after drug selection and identified through genomic PCR. Such methodology resulted in cells stably expressing DREADDs and mCherry under in vitro and in vivo conditions^35,40^. The hPSCs expressing DREADDs were next directed to differentiate into hMGE-like progenitors^23^. The hESCs were first directed to generate primitive neuroepithelial cells by incubating in a chemically defined neural induction medium for 10 days^23^. Following neural induction, cells were treated with sonic hedgehog (1000 ng/ml) for 2 weeks to pattern them into human medial ganglionic eminence (hMGE)-like progenitors. The identity of the hMGE-like cells in cultures was confirmed with NKX2.1 and FOXG1 immunostaining^23^.

### Transplantation of NKX2.1+ hMGE progenitors into hippocampi of rats with chronic TLE

The hMGE progenitors were transplanted ~35 days after neural induction. The neurospheres were dissociated into smaller cell aggregates and single cells, washed thoroughly by repeated centrifugation to remove dead cells, and >85% viable cells were obtained. For transplantation experiments, rats were anesthetized with an intramuscular injection of the anesthetic cocktail (a mixture of ketamine 50 mg/ml, xylazine at 4.5 mg/ml, and acepromazine at 0.4 mg/ml) at a dose of 0.7 ml/kg body weight. Approximately 100,000 viable cells in a 1.0 µl of differentiation medium were grafted into three sites in each hippocampus using a stereotaxic device and specific coordinates^22^. Cells were injected in 0.2 µl spurts over 5–8 min. Daily cyclosporine A injections (10 mg/kg) were given starting 2 days before transplantation and continuing until the experimental endpoint to avoid transplant rejection. Ungrafted control animals received similar cyclosporine injections throughout the survival period to rule out any cyclosporine-induced seizure regulation.

### Implantation of electrodes, EEG recordings, and analyses of SRS

Implantation of EEG electrodes was performed in the fourth month after transplantation. Each deeply anesthetized animal was fixed to a stereotactic device after clipping the hair over the head. Using aseptic procedures, a midline incision was made to the skin over the head, three burr holes were drilled in the skull, and sterilized metal EEG electrodes containing mounting screws were implanted epidurally. The electrodes comprised two recording electrodes over the frontoparietal cortices (one on each hemisphere) and a reference electrode over the cerebellum. Additional screws were placed over the frontal cortex to hold the dental cement. The electrode leads were micro plugged, and all electrodes and screws were cemented to the animal’s head^52^. After 2 weeks, rats were connected to a tethered video-EEG system and continuously monitored for simultaneously occurring behavior and electrographic activity in freely behaving rats. Baseline EEG recordings were taken for a week. Following this, CERs with grafts received injections of CNO (3 mg/Kg, once every 8 h for 2–3 days) to activate DREADDs. CERs with the CNO group also received CNO to test the effect of CNO alone on SRS activity. Baseline recordings were confirmed again for 4 days after the washout of CNO for 2 days. EEG tracings from CERs in all groups were analyzed for the frequency of all SRS, the frequency of stage-V SRS, and the percentage of time spent in seizure activity for the total recording period. Furthermore, 6–10 randomly selected 30-min interictal segments were analyzed in each animal for spectral density (*n* = 5 in pre-CNO, CNO, and post-CNO periods)^22^.

### Behavioral tests

Following EEG recordings, rats were probed for behavioral performance. An open field apparatus measuring 100 cm × 100 cm was employed for an object location test (OLT) and a pattern separation test (PST). The OLT began after a 7-day CNO washout period following EEG recordings (Fig. 1). Similarly, PST was initiated 7 days after the completion of OLT (Fig. 1). In OLT, the behavior of each rat was observed in an open field with three successive trials (T1–T3) separated by 15-min intervals. In the habituation phase (T1), each rat was acclimatized to the open field apparatus with no objects for 5 min. In the sample phase (T2), each rat was allowed to explore two identical objects placed in distant areas in an arena. In trial 3 or testing phase (T3), one of the objects was moved to a new area (object in novel place object, OINP) while the other object remained in the previous place (object in familiar place object, OIFP). Both T2 and T3 were video recorded using the Noldus–Ethovision video-tracking system to measure the amount of time spent with each of the two objects. Exploration of the object was defined as the length of time a rat’s nose was 1 cm away from the marked object area. The results such as the percentage of object exploration time spent exploring the OINP and OIFP and the total object exploration time in T3 were computed. The percentage of time spent with the OINP and OIFP was calculated by using the following formula: the time spent with the OINP/the total object exploration time × 100. Two days later, rats were injected with CNO for one hour, and the entire OLT was repeated with new objects for comprehending the impact of silencing the graft-derived GABA-ergic interneurons on object location memory.

The PST comprised four successive trials (T1–T4) with an inter-trial interval of 30-min. T1 involved acclimatization of the animal for 5 min in the open field apparatus. The animals explored a pair of identical objects (type 1 objects) placed in distant areas on a floor pattern (pattern 1 or P1) for 5 min in T2, whereas in T3, the animals explored the second pair of identical objects (type 2 objects) placed in distant areas on a different floor pattern (pattern 2 or P2) for 5 min. In T4, one of the T3 objects was replaced with an object from T2, which became a novel object on pattern 2 (NO on P2), whereas the object retained from T3 became a familiar object on P2 (FO on P2). The animal could explore objects for 5 min. Both T2 and T3 were video recorded using the Noldus-Ethovision video-tracking system. Object exploration was defined as the length of time a rat’s nose was 1 cm away from the object area. Time spent exploring the NO on P2 and the FO on P2 and the total object exploration time were computed from T4. Furthermore, the NO discrimination index was calculated using the following formula: the time spent with the NO on P2/the total object exploration time × 100. Two days later, rats were injected with CNO for 1 h, and the entire PST was repeated with new objects and floor patterns for understanding the impact of silencing the graft-derived GABA-ergic interneurons on pattern separation function.

### Brain tissue processing, immunohistochemistry, dual and triple immunofluorescence, and confocal microscopy

Deeply anesthetized rats were perfused with 4% paraformaldehyde, and the brains were removed and post-fixed in the same fixative for ~14 h. Following cryoprotection, 30-µm-thick coronal sections through the entire septotemporal axis of the hippocampus were collected serially in 24-well plates containing the phosphate buffer^52^. Immunohistochemical investigations comprised serial sections (every 15th or 20th) through the entire hippocampus^52^. Following etching in PBS containing 20% methanol and 3% hydrogen peroxide for 20 min, the sections were blocked with 10% serum for 30 min and incubated with the respective primary antibody solution for 16–24 h. The sections were then thoroughly washed in PBS and incubated successively in an appropriate biotinylated secondary antibody and avidin–biotin complex reagent for 60 min each. The peroxidase reaction was developed using Vector SG (Vector Lab, SK-4700) as the chromogen. After washing, the sections were mounted on gelatin-coated slides, dehydrated, cleared, and coverslipped with permount. For dual and triple immunofluorescence, the sections were blocked with 10% serum and incubated overnight in an individual or a cocktail of 2–3 primary antibodies raised in different species. Following incubation with secondary antibody conjugated with fluorescent markers, tissues were counterstained with DAPI and coverslipped in the slow fade–antifade medium.

The primary antibodies comprised mouse anti-human nuclear antigen (Millipore, MAB1281, 1:1000), mouse anti-NeuN (Millipore, MAB377, 1:1000), rabbit anti-GABA (Sigma, A2052, 1:5000), mouse anti-Parvalbumin (Sigma, P3088, 1:2000), Rabbit anti-Neuropeptide Y (Peninsula Laboratories, T-4070, 1:10,000), Goat anti-PSD95 (Abcam, ab12093, 1:500), Mouse anti-human Synaptophysin (ThermoFisher, 14-6525-80, 1:500), and Rabbit anti-MAP2 (Millipore, AB5622, 1:1000). Following secondary antibodies were used: Donkey anti-mouse AF 594, Invitrogen A-21203, 1:200), donkey anti-mouse AF 488 (Invitrogen, A21202, 1:200), Donkey anti-goat AF 488 (Invitrogen A11055,1:200), donkey anti-rabbit AF 488 (Invitrogen A21206, 1:200), donkey anti-rabbit AF 405, Abcam, ab175651, 1:200), Donkey anti Goat Cy3, (Jackson ImmunoResearch, 705-166-147, 1:200), Goat anti-mouse AF 488, (Invitrogen A 32723, 1:200), Goat anti-mouse AF594, (Invitrogen A 11032, 1:200). For phenotypic analyses of graft-derived neurons using dual and triple immunofluorescence methods, 1.5 µm thick optical *Z*-sections were collected from 6 randomly selected graft regions in each rat. The images were analyzed using the NIS image browser^22,56^. The confocal images were adjusted for brightness and contrast in Photoshop. Immunostaining spanning over three successive *Z*-sections was considered positive staining.

### Stereological quantification of HNA+ cells

The number of HNA+ cells per hippocampus was measured using an optical fractionator method in a StereoInvestigator system (Microbrightfield Inc., Williston, VT, USA) comprising a digital video camera (Optronics Inc., Muskogee, OK, USA) interfaced with a Nikon E600 microscope^57^. In each animal, HNA+ cells were counted from 62–76 frames (each measuring 20 × 20 µm, 0.0004 mm^2^ area) in every 15th section through the entire hippocampus using the 100x oil immersion objective lens. In every section, the contour of the hippocampus was first marked using the tracing function. The number, the location of counting frames, and counting depth were determined by entering parameters such as the grid size (600 × 600 µm), the thickness of the guard zone (4 µm), and the optical dissector height (8 µm). A computer-driven motorized stage then allowed the section to be analyzed at each counting frame location. In each location, the top of the section was set, and the plane of the focus was moved 4 µm deeper through the section (guard zone) to reach the first point of the counting process. Continuing to focus, any HNA+ cells that came into focus in the next 8-µm section thickness were counted if they were entirely within the counting frame or touching the upper or right side of the counting frame. By utilizing cell counts generated as above and the optical fractionator formula, the StereoInvestigator program calculated the total number of HNA+ cells per hippocampus^57^. The average Gundersen CE was 0.135 in these cell counts (Mean ± S.E.M = 0.135 ± 0.032, *n* = 4).

### Statistical analysis

Statistical analyses were performed using GraphPad Prism software. Unpaired, two-tailed Student’s *t* tests were employed when two groups were compared. One-way ANOVA with Newman–Keuls multiple comparison post hoc tests was employed when three or more groups were compared. Numerical data were presented as mean ± S.E.M., and *p* < 0.05 was considered statistically significant.

### Reporting summary

Further information on research design is available in the Nature Research Reporting Summary linked to this article.

## Supplementary information

REPORTING SUMMARY

## Data Availability

All data needed to evaluate the results of this article are present in the paper.
