# Supplementary material for: Grafted hPSC-derived GABA-ergic interneurons regulate seizures and specific cognitive function in temporal lobe epilepsy
Source: NPJ Regen Med. 2022 Aug 1;7:38. doi: 10.1038/s41536-022-00234-7 (PMC9343458; doi:10.1038/s41536-022-00234-7)
Supplement: Supplementary file 1 — REPORTING SUMMARY [file 41536_2022_234_MOESM1_ESM.pdf]

nature portfolio

Corresponding author(s): DBPR: NPJREGENMED-00792R.

Last updated by author(s): 05/19/2022

Reporting Summary

Nature Portfolio wishes to improve the reproducibility of the work that we publish. This form provides structure for consistency and transparency in reporting. For further information on Nature Portfolio policies, see our Editorial Policies and the Editorial Policy Checklist.

Statistics

For all statistical analyses, confirm that the following items are present in the figure legend, table legend, main text, or Methods section.

n/a	Confirmed	
☐	☒	The exact sample size (*n*) for each experimental group/condition, given as a discrete number and unit of measurement
☐	☒	A statement on whether measurements were taken from distinct samples or whether the same sample was measured repeatedly
☐	☒	The statistical test(s) used AND whether they are one- or two-sided<br>*Only common tests should be described solely by name; describe more complex techniques in the Methods section.*
☒	☐	A description of all covariates tested
☐	☒	A description of any assumptions or corrections, such as tests of normality and adjustment for multiple comparisons
☒	☐	A full description of the statistical parameters including central tendency (e.g. means) or other basic estimates (e.g. regression coefficient) AND variation (e.g. standard deviation) or associated estimates of uncertainty (e.g. confidence intervals)
☐	☒	For null hypothesis testing, the test statistic (e.g. *F*, *t*, *r*) with confidence intervals, effect sizes, degrees of freedom and *P* value noted<br>*Give P values as exact values whenever suitable.*
☒	☐	For Bayesian analysis, information on the choice of priors and Markov chain Monte Carlo settings
☒	☐	For hierarchical and complex designs, identification of the appropriate level for tests and full reporting of outcomes
☒	☐	Estimates of effect sizes (e.g. Cohen's *d*, Pearson's *r*), indicating how they were calculated

Our web collection on statistics for biologists contains articles on many of the points above.

Software and code

Policy information about availability of computer code

Data collection	StereoInvestigator software from Microbrightfield LLC was used for cell counts.
Data analysis	GraphPad Prism software was used for statistical anlayses.

For manuscripts utilizing custom algorithms or software that are central to the research but not yet described in published literature, software must be made available to editors and reviewers. We strongly encourage code deposition in a community repository (e.g. GitHub). See the Nature Portfolio guidelines for submitting code & software for further information.

Data

Policy information about availability of data

All manuscripts must include a data availability statement. This statement should provide the following information, where applicable:
- Accession codes, unique identifiers, or web links for publicly available datasets
- A description of any restrictions on data availability
- For clinical datasets or third party data, please ensure that the statement adheres to our policy

All data needed to evaluate the results of this article are present in the paper.

Field-specific reporting

Please select the one below that is the best fit for your research. If you are not sure, read the appropriate sections before making your selection.

☒ Life sciences ☐ Behavioural & social sciences ☐ Ecological, evolutionary & environmental sciences

For a reference copy of the document with all sections, see nature.com/documents/nr-reporting-summary-flat.pdf

Life sciences study design

All studies must disclose on these points even when the disclosure is negative.

Sample size	The sample size was determined based on our previous studies for similar experiments. The sample sizes chosen in the study were sufficient based on the demonstrated statistical significance between groups.
Data exclusions	No data were excluded
Replication	The reproducibility of the experimental design is apparent from our findings on seizures. In the study, grafting of human interneuron progenitors into the brain reduced seizures in the temporal lobe epilepsy model but blocking the activity of graft-derived interneurons using a designer drug resulted in increased seizures. Furthermore, following the washout of the designer drug, the seizures were reduced again. Also, the ability of human interneuron progenitors to reduce seizures in the temporal lobe epilepsy model is consistent with our previous study in the status epilepticus model (Upadhya et al., PNAS, 2019).
Randomization	Chronically epileptic rats having a similar range of seizures were randomly assigned to different groups; one group received grafts expressing designer receptors and designer drug treatment, the second group received no grafts, and the third group received only the designer drug.
Blinding	Investigators who quantified EEG traces were blinded to group allocations.

Reporting for specific materials, systems and methods

We require information from authors about some types of materials, experimental systems and methods used in many studies. Here, indicate whether each material, system or method listed is relevant to your study. If you are not sure if a list item applies to your research, read the appropriate section before selecting a response.

Materials & experimental systems

n/a	Involved in the study	
☐	☒	Antibodies
☒	☐	Eukaryotic cell lines
☒	☐	Palaeontology and archaeology
☐	☒	Animals and other organisms
☒	☐	Human research participants
☒	☐	Clinical data
☒	☐	Dual use research of concern

Methods

n/a	Involved in the study	
☒	☐	ChIP-seq
☒	☐	Flow cytometry
☒	☐	MRI-based neuroimaging

Antibodies

Antibodies used	Mouse anti-human nuclear antigen (Millipore, MAB1281), mouse anti-NeuN (Millipore, MAB377), rabbit anti-GABA (Sigma, A2052), mouse anti-Parvalbumin (Sigma, P3088), Rabbit anti-Neuropeptide Y (Peninsula Laboratories, T-4070). Goat anti-PSD95 (Abcam, ab12093); Mouse anti-human Synaptophysin (ThermoFisher, 14-6525-80), Rabbit anti-MAP2 (Millipore, AB5622).
Validation	All antibodies were validated using positive control and negative control brain tissue sections before employing them in the study.

Animals and other organisms

Policy information about studies involving animals; ARRIVE guidelines recommended for reporting animal research

Laboratory animals	Male Fischer 344 rats
Wild animals	N/A
Field-collected samples	N/A
Ethics oversight	All experiments were performed as described in the animal protocol, approved by the institutional animal care and use committee of the Texas A&M Health Sciences Center and Central Texas Veterans Health Care System.

Note that full information on the approval of the study protocol must also be provided in the manuscript.
